# Supplementary material for: Patterns of Intron Gain and Loss in Fungi
Source: PLoS Biol. 2004 Nov 30;2(12):e422. doi: 10.1371/journal.pbio.0020422 (PMC532390; doi:10.1371/journal.pbio.0020422)
Supplement: Table S1 — Also available at http://genes.mit.edu/NielsenEtAl/. (4.3 MB ZIP). [file pbio.0020422.st001.zip › NielsenEtAl/html/954.html]

AN6394.1.NCU08924.1.MG05949.1.FG10285.1


```
 CLUSTAL W (1.82) Multiple Sequence Alignments - Introns Inserted


Sequence 1: NCU08924.1	519 aa
Sequence 2: MG05949.1	513 aa
Sequence 3: FG10285.1	512 aa
Sequence 4: AN6394.1	512 aa
Alignment Length: 521 aa
Number Identitical Residues: 282 aa
Alignment Score (without introns) 14146


MG05949.1 	-----MASAKTFTREEVKKHNTEDDVWFIIDSVVYDVSDFLDAHPGGEAVLRQAAGTDAT
NCU08924.1	MTNVLNVPDKTFTREEVKKHNTEDSTWFIIDSVVYDVSEFLDAHPGGEAVLRQVAGQDAT
FG10285.1 	-------MTDTFSREEVRSHTSDESLWCIIDSTVYDLTDFVDAHPGGETVLRQVAGQDAT
AN6394.1  	-------MSKTFTRDEVAKHNTEDSLWCIIDHRVYDLTDFLDAHPGGSVVLSQIAGKDAT
          	         .**:*:** .*.:::. * ***  ***:::*:******..** * ** ***

MG05949.1 	TDFYNLHRHEV2LTKYKD-LAIGTIVNEKPSVITPKPGDLSLVPYAEPLWLTPQFKSPYY
NCU08924.1	SAFYNLHRHEV~LIKYND-LAIGSIKGEKQSIITPRPGDLSPVPYAEPLWLTPQFKSPYF
FG10285.1 	TAFYNLHRHEV~LTKYKD-LAVGTIEGEKAQVITPQPGDLSQVPYAEPLWLAEPFRSPYY
AN6394.1  	TDFYNLHRHEV~LTKYRDSLCIGTIADEKPEVIDPTPDGLSPVPYAEPLWLRPGFKSPYY
          	: ********* * **.*:*.:*:* .** .:* * *..** *********   *:***:

MG05949.1 	KESHHRLRRAMREFTDIYTTPEAQAREKDGEYVSQELVDRMSKAGILHMRMGPGKHLHGQ
NCU08924.1	NDSHRSLQKAIRKFTDEVIAPEALEKERTGELISQDLIDKMSKAGILHMRMGPGKHLHGV
FG10285.1 	KDSHRELRRKLREFVDSELYEEAQECEATGRYISQKMIDRMSELGILHMRLGPGKHLHGV
AN6394.1  	NESHRRLQKAIREFTSLHVAPEAAEKEKDGTYISQELINKMAETNVLAMRLGPGKHLHGR
          	::**: *:: :*:*..     **   *  *  :**.::::*:: .:* **:******** 

MG05949.1 	NLLDGAVDGKEFDYFHDLICVQEQTRPSARG~FQDGNLAGMVIGLTAVLNFCKDEP-LKK
NCU08924.1	NLLDGAVKGEEFDYFHDMIMAQEQVRVACRG~FQDGNMAGMAIGLTCILNYGDKNPALKK
FG10285.1 	NLMGGAVKGEEFDYFHDLIVSQELARAMARG~FADGNMAGMTISLTAVLNFARDEA-WKN
AN6394.1  	KLLGGVVDGKEFDYLHDLIIAQEMVRVNARG1FQDGNMAGMVISLTAVQNWLRNEP-LRQ
          	:*:.*.*.*:****:**:*  ** .*  .** * ***:***.*.**.: *:  .:.  ::

MG05949.1 	RITDEVFSGKKKICLAITEAFAGSDVANLRTTAKKTADGKHY~IVNGTK~KWITNG1VFC
NCU08924.1	RITEEVFAGKKKICLAITEAFAGSDVAGLRTTATKTPDGKHY~IVNGTK~KWITNG~VFC
FG10285.1 	KIANEVFSGKKKICLAITEAFAGSDVAGLRTTAEKTPDGKHY0IINGTK~KWITNG~VWC
AN6394.1  	KVTEEVLSGKKKMCLAVTEAFAGSDVAGLRTTAEKTPDGKYY~IVNGTK2KWITNG~MYA
          	::::**::****:***:**********.***** **.***:* *:**** ****** ::.

MG05949.1 	DYFVAGVQTDKGLSVLIIERGEGVETQQMKTSYSRTAGTTYVTFDNVKVPVGHLLGEENK
NCU08924.1	DYFVTGVKTDKGLSVLLIERGEGVETKPIKTSYSPAAGTTYITFDNVKVPVENLLGEENK
FG10285.1 	DYFVTGARTDKGLSVFLIERGEGVETKQIKTSYSTTAGTAYVTFDNVKVPAENMLGKENK
AN6394.1  	DYFVTACRTSKGFSVLLIPRDSAVETTQIKTSYSTTAATAFVEFNNVKVPVDHLLGEEHK
          	****:. :*.**:**::* *...***  :***** :*.*::: *:*****. ::**:*:*

MG05949.1 	GIYVILSNFNHERWTMTC~GTVRYCRTIVEECLKWAHQRLVFGKRLIDQPVIRLK~LAKM
NCU08924.1	GIYVILSNFNHERWMMAC~ATIRYMRLVVEESLKWSHQRLVFGKRLIDQPVIRQK~LAKM
FG10285.1 	GIQVILSNFNHERWTMVC1GSLRLSRSIVEECLKWANQRQVFGKSLIDQPVIRQK2LAKM
AN6394.1  	GFIVVMSNFNHERYMMVC~AVVRFSMTVVEECLKWCNQRIVFGKRLIEQPVIRQK~LARM
          	*: *::*******: *.* . :*    :***.***.:** **** **:***** * **:*

MG05949.1 	IALVESHQSWLETIT~YQMCHMPYSEQSKHLGGPIGLLKMSATRMAHEIADESVQIWGGR
NCU08924.1	IALCESHQSWLETIT2YQMNHMPYSEQAKHLGGPIALLKMSCTRAAHEIADESVQIWGGR
FG10285.1 	IALVEANQSWLETIT~YQMCHMPYSQQAQHLAGPIGLLKMSATRAAHEVADESVQIWGGR
AN6394.1  	ISLTESNQAWLESIA~YQMNNMTYAEQSKNLGGPIGLLKSHVTRCAGEIAEQATNIFGGR
          	*:* *::*:***:*: *** :*.*::*:::*.***.***   ** * *:*:::.:*:***

MG05949.1 	GLTQTGMGYIIENFNRTYKFDAIL0GGAEEVLGDLGVRQAMKNMPKAML~-
NCU08924.1	GLTQTGMGRVIENFNRGYKFDAIL~GGAEEVLGDLGVRQAMKFMPKAVL~-
FG10285.1 	GITQTGMGKFIEMFHRTYKFDAIL~GGAEEVLGDLGVRQAMRNFPNAKF2I
AN6394.1  	GLTQTGMGRVIEMFNRTYKFDSIL~GGTEEILADLGVRQAYKSFPKAML~-
          	*:****** .** *:* ****:** **:**:*.******* : :*:* :
```
